# Tunable Wood by Reversible Interlocking and Bioinspired Mechanical Gradients

**DOI:** 10.1002/advs.201802190

**Published:** 2019-03-28

**Authors:** Marion Frey, Giulia Biffi, Maria Adobes‐Vidal, Meri Zirkelbach, Yaru Wang, Kunkun Tu, Ann M. Hirt, Kunal Masania, Ingo Burgert, Tobias Keplinger

**Affiliations:** ^1^ Wood Materials Science Department of Civil, Environmental and Geomatic Engineering ETH Zürich 8093 Zürich Switzerland; ^2^ Cellulose & Wood Materials Functional Materials EMPA 8600 Dübendorf Switzerland; ^3^ Design and Arts Lucerne University of Applied Sciences and Arts 6020 Emmen Switzerland; ^4^ Institute for Geophysics Department of Earth Sciences ETH Zürich 8093 Zürich Switzerland; ^5^ Complex Materials Department of Materials ETH Zürich 8093 Zürich Switzerland

**Keywords:** delignification, mechanical gradients, natural fiber composites, reversible mechanical interlocking, shapeable wood

## Abstract

Elegant design principles in biological materials such as stiffness gradients or sophisticated interfaces provide ingenious solutions for an efficient improvement of their mechanical properties. When materials such as wood are directly used in high‐performance applications, it is not possible to entirely profit from these optimizations because stiffness alterations and fiber alignment of the natural material are not designed for the desired application. In this work, wood is turned into a versatile engineering material by incorporating mechanical gradients and by locally adapting the fiber alignment, using a shaping mechanism enabled by reversible interlocks between wood cells. Delignification of the renewable resource wood, a subsequent topographic stacking of the cellulosic scaffolds, and a final densification allow fabrication of desired 3D shapes with tunable fiber architecture. Additionally, prior functionalization of the cellulose scaffolds allows for obtaining tunable functionality combined with mechanical gradients. Locally controllable elastic moduli between 5 and 35 GPa are obtained, inspired by the ability of trees to tailor their macro‐ and micro‐structure. The versatility of this approach has significant relevance in the emerging field of high‐performance materials from renewable resources.

Biological materials are able to optimize their structure^1^, ^2^ and chemical composition^3^ to adapt to changing environmental conditions.^4^ They can cope with external loading conditions by locally altering their stiffness, for example by adjusting the density,^2^ the alignment of stiff reinforcing building blocks,^5^, ^6^ the chemical constituents,^3^ or by interface design strategies.^7^ Interfaces in biological materials can rely on structure or chemistry. In the example of wood, the interface properties between stiff cellulose fibrils and the soft matrix are determined by a multitude of weak chemical bonds,^8^ whereas in the beak of the red‐bellied woodpecker^9^ or in the osteoderms of a leatherback sea turtle shell, jigsaw‐like or sutured patterned interfaces lead to stress transfer or enable deformation.^10^

The efficient design of biological materials comprising hierarchical structuring, gradients, functionality, and specific interface structures has been role model for the development of bioinspired materials.^11^ Although various bottom‐up approaches have shown the potential of assembling building blocks to transfer bioinspired design principles into synthetic materials,^12^ it still remains challenging to reach the structural complexity of biological materials and to fabricate them in an environmentally‐friendly and scalable manner.^4^ In contrast, when biological materials such as wood are used in top‐down approaches, their hierarchical structure can be retained and modified.^13^ A top‐down wood modification approach gaining increasing attention is the delignification of wood by a structure‐preserving treatment, which leads to formability and enhanced mechanical performance upon densification of the material.^14^, ^15^ However, the full potential of cellulose scaffolds has not yet been exploited. Beyond rather simple densification treatments, the delignified wood could be processed further to obtain a novel material combining adaptation of shape, mechanical gradients, and tunable functionality.

Here we present a versatile strategy to develop smart materials of renewable nature and enhanced performance based on wood by implementing bioinspired structural, chemical, and mechanical gradients as well as adaptive shaping. We tune mechanical properties and locally optimize the material for external loading conditions in a simple, scalable, and predictable manner. A moisture‐triggered formability is induced by removing the lignin interface between cell walls, which enables shear deformation of the material in wet state. This type of shaping results in an optimized fiber orientation, mimicking the adaptive fiber alignment in trees.^6^, ^16^ Densification and drying causes close contact between fibers and creates strong interfaces based on mechanical interlocking and hydrogen bonding. By locally varying the degree of densification, we show the ability to spatially tune the mechanical and functional properties. A superhydrophobic surface treatment of the desired geometry finally freezes the obtained architecture. With our approach biological design principles, such as density gradients, fiber alignment, and reversible interlocking mechanisms, are incorporated into the hierarchical wood material.

Structure preserving delignification of wood results in the removal of the matrix lignin, which acts as an adhesive between the wood fibers (tracheids). Remarkably, delignified wood is still mechanically stable in its dry state at 20 °C/65% RH with a water content of 11.1%. The stability is due to mechanical interlocking in the cell corner regions, which leads to connected neighboring fibers and compensates for the removed lignin matrix. In this state, delignified wood can be used as a structural material. In contrast, in the water‐saturated morphing state (water content of 435.4%), water disengages the interlocks by swelling the material and creating space between the tracheids, which leads to a low shear modulus, making the wood shapeable (**Figure** **1**).

**Figure 1:**
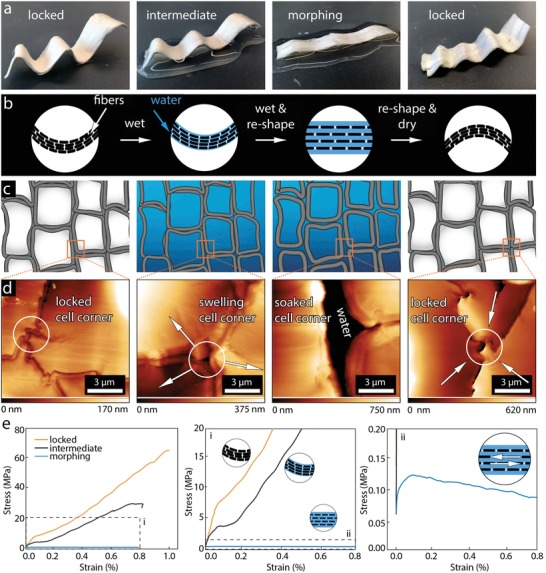
Shaping mechanism of delignified wood using moisture induced reversible interlocks. a) Delignified wood samples in the locked, intermediate, morphing, and re‐locked (after reshaping) state. b) Scheme of a longitudinal section of delignified wood in the four states, illustrating the swelling of delignified wood fibers (black) by water (blue). c) Scheme of a cross section and d) AFM images of the cell corner region showing the moisture induced interlocking mechanism. e) Representative tensile stress–strain curves of delignified wood in the locked (orange), intermediate (black), and morphing state (blue) and zoom into regions of interest (i,ii).

To investigate the water‐triggered shaping of the cellulosic material on a macroscopic scale, a delignified wood veneer was shaped using a mold characterized by varying radii of curvature. When the obtained shape was rewetted, softening of the delignified wood, caused by the decreased interaction between neighboring fibers, allowed for a repeated shaping of the material (Figure 1a,b).

In order to gain a detailed insight into the underlying mechanisms, microscopic investigation by AFM imaging was conducted. In the dry state, the cell walls of the fibers are in close contact to one another, shown schematically in Figure 1c. In the former cell corner regions, shrinkage resulted in a sawtooth‐like interlocking pattern between neighboring cell walls (Figure 1d). This tight interlocking combined with frictional forces and hydrogen bonding between OH‐groups of the cellulose fibers leads to a stiff and strong material, even in the absence of lignin. The morphology was found to gradually change with increasing water content. First, water absorption in the cell walls results in a swelling and regaining of their initial round shape (intermediate state). In the wet state, free water accumulates between fibers as shown by in situ AFM images (Figure 1d). Softening of the cellulosic scaffold upon water uptake is further shown by the corresponding mechanical properties. Tensile tests performed on 5 mm thick delignified bulk wood samples revealed an elastic modulus of 6.5 GPa (Figure 1e, orange curve) in the dry state. When testing samples at the intermediate state with a water content of 22.9%, an elastic modulus of 3.8 GPa (black curve) was measured. This can be explained by the onset of swelling accompanied by decreased interactions between the cells. In the wet morphing state, water allows for a strong plastic formability with very little stress build up at increasing levels of strain, shown by the tensile curve in blue. The decrease in strength after reaching a maximum value of 125 Pa corresponds to a slip‐off mechanism between fibers, which is crucial for successful shaping of the material.

The shear deformation mode between de‐coupled neighboring fibers is visualized by bending of a delignified bulk wood sample as illustrated in **Figure** **2**a. A bending deformation of perfectly coupled fibers would result in an edge orthogonal to the neutral axes, which was not observed for the delignified wood sample. Instead, vertically aligned edges demonstrate that there is low interfacial shear transfer between the fibers, which allows the wood cells to easily slide past one another. Considering the importance of a sufficient de‐coupling of neighboring fibers to allow shear deformation of the material, the role of remaining lignin after delignification has to be investigated. Partially delignified wood molded in the same manner as shown in Figure 2b, was not formable to the curvatures (radii between 200 µm and 7 mm) of the mold, shown in Figure 2c–e. The fibers were observed to wrinkle and rupture upon shaping, being especially apparent in the latewood regions of the sample. This can be explained by the higher lignin content remaining in the latewood after partial delignification compared to earlywood (Figure S1, Supporting Information), which strongly affects the shear modulus of the wood. This rupturing could be observed on both, macroscopic scale and microscopic scale (Figure 2c–e).

**Figure 2:**
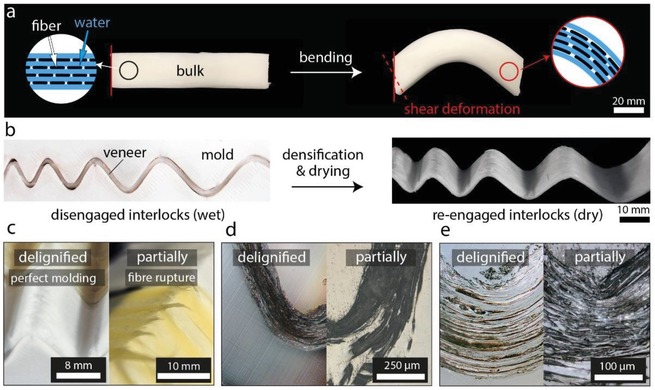
Bending of a delignified bulk wood sample in the morphing state results in a) a shear deformed, curved sample. The sample shows vertically aligned edges (red line) after bending. The red dotted line illustrates the orthogonal to the neutral axes that would be expected for perfectly bonded interfaces. b) Densification of a delignified veneer in a cast ceramic mold followed by drying results in a stiff material due to the locked cell corners. c) Wet delignified wood drapes onto the mold while partially delignified wood veneers wrinkle and rupture. d,e) Light‐microscope images of delignified and partially delignified veneers after densification in the mold demonstrate that complete removal of lignin is required to disengage mechanical interlocking of cell walls.

The mechanical properties of delignified wood were adjusted by varying the density locally, which directly impacted the stiffness and strength behavior of the material. To measure the stiffness and strength of the material at different densities, tensile tests were conducted on densified cellulose materials with fiber volume contents (FVCs) between 20% and 85%. Thin delignified bulk wood samples with matching growth ring positions were stacked and densified in radial direction as shown in **Figure** **3**a and the FVC was controlled by the number of used layers and by the resulting thickness after densification. Of note, the achieved values of 85% FVC are far beyond the typical 65% FVC of continuous fiber‐reinforced composites and in contrast to the later, no polymeric matrix was needed, as the wood cells were mechanically interlocked.

**Figure 3:**
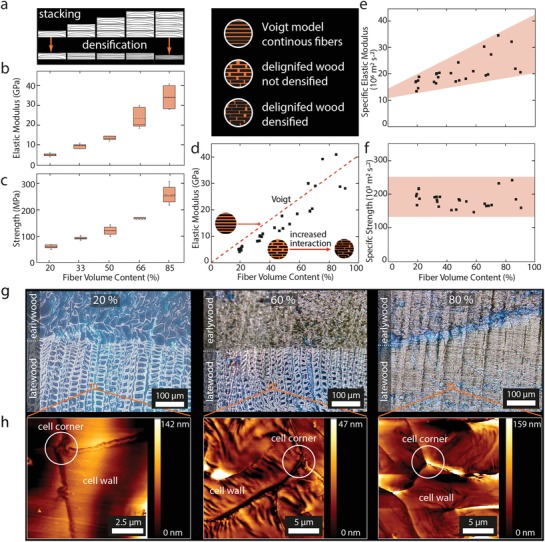
a) Stacking results in FVCs between 20% before densification up to values of 85% upon densification. b) Tensile elastic modulus and c) strength versus normalized FVC demonstrate the broad range of properties that can be achieved. d) Elastic moduli compared to the Voigt model. e) Specific elastic modulus and f) specific strength for FVCs between 20% and 85%. g) Light‐microscope images show the densification behavior of delignified early‐ and latewood at 20%, 60%, and 80% FVC. h) AFM images on cell wall level of 20%, 60%, and 80% FVC samples show notable wrinkling at FVCs above 60%.

Depending on the FVC, a stiffness between 5 and 35 GPa (Figure 3b) and corresponding strengths between 60 and 270 MPa (Figure 3c) were obtained. A linear increase in stiffness and strength with increasing fiber volume content has been observed until 60%, which is a typical behavior for continuous fiber‐reinforced materials in axial loading, described by the Voigt model (Figure 3d). Despite the delignified wood being a short fiber‐reinforced material, the slope corresponds to the Voigt model, assuming an elastic modulus of 40 GPa for single wood fibers.^17^ Hence, in the dry state the close contact between the fibers combined with mechanical interlocks and hydrogen bonding allows for excellent stress transfer, resulting in a continuous fiber‐reinforced material‐like behavior in this matrix‐free wood composite.

Light‐microscopy and AFM imaging were used to analyze the densification process of the stacked samples on a tissue‐ and fiber level. A densification up to FVC of 60% mainly results in a densification of the earlywood (Figure 3g). At this densification level, the latewood was not yet compressed, but rather started to wrinkle on the cell wall scale, as seen in the AFM images (Figure 3h). At 60% FVC, latewood cells are slightly deformed and show a transition to a completely densified state at 80% FVC. Interestingly, at a FVC of 80%, a higher than expected increase of the elastic modulus is observed. This suggests an improvement of mechanical properties, which cannot solely be attributed to the increased FVC and is supported by the increase of specific elastic modulus with FVC. Possible contributions to these phenomena could include increased hydrogen bonding interactions and additional friction forces, enabled through the close contact between fibers. Additionally, enhanced mechanical interlocking and entanglement between fibers caused by the densification could contribute to increased stiffness. In contrast, at a FVC of 85%, lower values than the predicted Voigt model were obtained for the elastic modulus. This is likely explained by difficulties in the manufacturing process of samples with FVC above 85%, which resulted in deviations in the unidirectional fiber arrangement and potential damage to the cellulose fibers within the samples.

A local variation of the FVC within one sample by topographic stacking leads to mechanical gradients and can be combined with other tree‐inspired concepts such as adjusting the fiber alignment to reduce stress concentrations.^18^, ^19^ The demonstration of an open hole structure shown in **Figure** **4**a illustrates the possibility of combining both, fiber alignment and density gradients in one manufacturing process. The fiber alignment of the delignified veneer was pre‐programmed by simply shaping the material in wet state. This mimics design principles that are common to native wood structures such as a branch attachment on the macroscopic scale^19^, ^20^ or the adjusted fiber orientation around wood rays on the microscopic scale as shown in Figure S2, Supporting Information. Additional delignified veneers could be placed and densified at highly loaded regions, providing local reinforcement by in‐plane density gradients as illustrated in **Figure** **5**a. Figure 5b shows a topographically stacked 0/90˚ lay‐up material with layers increasing from 2 (left side) to 5 (right side), resulting in a density gradient. Alternatively, in plane mechanical gradients were obtained through local densification causing continuous density gradients with reduced thickness in the highly‐densified regions (Figure 5c,d).

**Figure 4:**
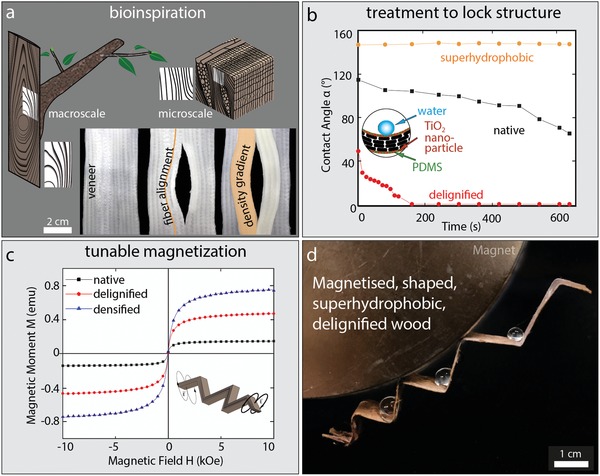
a) Illustration of fiber alignment and density gradients in a wood ray and in a tree branch attachment to the trunk. The concept of fiber alignment is transferred to the delignified, densified wood for a prescribed load path by a combination of tuned reinforcement, laminate stacking, and gradient densification. b) The wood was treated by a TiO_2_/PDMS coating to obtain delignified‐superhydrophobic wood, allowing the material to remain robust against moisture ingression for usage under wet conditions. c) Native, delignified, and delignified/densified wood was magnetized to demonstrate the possibility to gain enhanced functionality by using delignified and densified wood compared to native wood. d) Shaped, magnetic, superhydrophobic delignified wood as a proof of concept.

**Figure 5:**
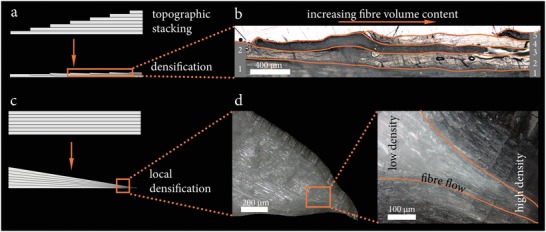
a) Scheme of the topographic stacking method followed by densification. b) Light‐microscopy image of a topographically stacked material. The density is increased from left (two layers) to right (five layers). The layers are stacked 0/90° in order to distinguish the layers optically from each other. c) Scheme of the local densification method. A stacked sample is densified into a wedge, which results in an increased density toward the tip. d) Light‐microscopy image (longitudinal cut) of delignified wood densified into a wedge.

While formability of the material in its wet state is a major advantage, when being used for load‐bearing scenarios, the strong decrease in mechanical performance under wet conditions prevents structural applications. The desired shape could be permanently kept in the interlocked state by superhydrophobizing the surface as shown in Figure 4b, which allows for continued integrity of the structure due to repellence of water. A delignified veneer was dip‐coated with a suspension of TiO_2_ nanoparticles dispersed in PDMS/THF providing a hydrophobic coating with high abrasive resistance.^21^ SEM imaging (Figure S3, Supporting Information) revealed a homogeneous coverage of the hydrophobic TiO_2_ nanoparticles on the surface of the delignified wood. Contact angle measurements were performed in order to investigate the performance of the coating. For uncoated native wood, the static contact angle (115°) decreased with time, as water started to penetrate into the hydrophilic surface. For the delignified samples, the initial contact angle of 50°, is about 50% less than native wood and decreases over time in an even more pronounced manner due to the removal of the hydrophobic lignin. On the PDMS and TiO_2_ treated surface however, the initial contact angle of approximately 150° remains constant with time (Figure S4, Supporting Information).

To demonstrate the possibility of combining mechanical gradients with tunable functionality, magnetite (Fe_3_O_4_) and maghemite (γ‐Fe_2_O_3_) were in situ synthesized inside native and delignified wood by co‐precipitation of ferric and ferrous salts (Figures S5, S6, Supporting Information). The precipitated particles carry a net magnetization in the presence of an external field. The delignified magnetic wood showed significantly higher values for saturation magnetization (Figure 4c) and susceptibility (Figure S7b, Supporting Information) compared to native magnetic wood. Upon densification in the radial direction, the magnetic moment and the susceptibility were further increased. Magnetic moment values of 1.2, 3.6, and 6 emu cm^−3^ were obtained for the native wood, delignified wood, and the densified delignified wood, respectively. A similar trend was observed for the susceptibility with a pronounced anisotropic behavior due to the intrinsic microstructure of wood (Figure S7b, Supporting Information). SEM imaging revealed the reason for the difference in magnetization between delignified and native functionalized wood. Iron‐oxide nanoparticles were mainly in the lumen of the native functionalized wood (Figure S7c,d, Supporting Information) and functionalization was not observed at the interface between neighboring fibers (middle lamella), which indicates that the infiltration of the precursor solution mainly takes place via the lumen of the cells. Contrary, the magnetic delignified wood additionally showed nanoparticles in the interface regions between neighboring fibers (Figure S7d, Supporting Information). This suggests that the precursor solution was able to not only infiltrate through the lumen, but also via the free space created between neighboring fibers through swelling. Finally, topographic stacking of magnetized delignified wood, followed by a densification in a shaped mold, led to tunable wood comprising bioinspired fiber alignment combined with mechanical and functional gradients (Figure 4d).

In conclusion, wet shaping of delignified wood and the implementation of structural and chemical gradients allows for locally tuning shape and mechanical properties in a predictable manner, which offers the possibility to turn natural wood into a versatile engineering material with excellent mechanical performance. The shaping mechanism is based on moisture triggered reversible interlocking between neighboring cells, which enable shear deformation in the wet state. Upon drying, mechanical interlocks and hydrogen bonding result in a stiff structural material, which can be further enhanced upon densification. Mechanical gradients were created by simply adjusting the density of the material to obtain very high FVCs up to 85%, and elastic moduli of 35 GPa at 270 MPa strength. The concept was further extended by using magnetically functionalized delignified wood, leading to tunable functionality across individual samples by adjusting the density. Additionally, a surface hydrophobization can protect the matrix‐free wood from liquid water uptake. This approach demonstrates the possibility to create mechanical gradients with tunable functionality in wood and has broad relevance in the emerging field of sustainable high‐performance materials.

## Experimental Section

*Delignification*: Norway spruce samples were delignified following the previously established protocol published in Frey et al., 2018 and Segmehl et al., 2018.^14^ The reaction time was adjusted according to the sample dimensions and is summarized in Table S1, Supporting Information. After delignification, all samples were washed with deionized water until the washing solution reached a pH value above 5 and were conditioned at 65% RH/20 °C until a constant mass was obtained.

*Preparation of Tensile Test Specimens*: Specimens with fiber volume contents (FVC) between 20% and 85% were prepared by densification (see Supplementary methods). Beech tags with an angle of 45° were glued to the grip section of thin bulk samples. Prior to mechanical testing the samples were conditioned in three different environments: 65%, 95% relative humidity, and water saturation.

*Shaping of Delignified Veneers*: Delignified wet veneers (*t* = 0.9 and 1.5 mm) were shaped and densified in closed molds with varying radius of curvature followed by vacuum drying. Molds were manufactured in two steps. First, the negative form was 3D printed with an Ultimaker 2+ (Ultmaker, Netherlands) and 1.75 mm thick PLA filaments (BQ, Spain). In a second step, the cast ceramic (Giluform, Suter Kunststoffe AG, Switzerland) was poured into the PLA form to obtain the positive mold.

## Conflict of Interest

The authors declare no conflict of interest.

## Supporting information

Supplementary

## References

[1] S. E. Naleway , M. M. Porter , J. McKittrick , M. A. Meyers , Adv. Mater. 2015, 27, 5455. 26305858 10.1002/adma.201502403

[2] a) M. A. Kasapi , J. M. Gosline , J. Exp. Biol. 1997, 200, 1639; 9202450 10.1242/jeb.200.11.1639

[3] a) J. H. Waite , H. C. Lichtenegger , G. D. Stucky , P. Hansma , Biochemistry 2004, 43, 7653; 15196007 10.1021/bi049380h PMC1839050

[4] Z. Liu , M. A. Meyers , Z. Zhang , R. O. Ritchie , Prog. Mater. Sci. 2017, 88, 467.

[5] I. Burgert , K. Frühmann , J. Keckes , P. Fratzl , S. Stanzl‐Tschegg , Trees 2004, 18, 480.

[6] C. Mattheck , Design in Nature. Trees as Instructors, Rombach Verlag, Freiburg im Breisgau 1997.

[7] a) J. W. Dunlop , R. Weinkamer , P. Fratzl , Mater. Today 2011, 14, 70;

[8] P. Fratzl , I. Burgert , H. S. Gupta , Phys. Chem. Chem. Phys. 2004, 6, 5575.

[9] N. Lee , M. Horstemeyer , H. Rhee , B. Nabors , J. Liao , L. N. Williams , J. R. Soc., Interface 2014, 11, 20140274. 24812053 10.1098/rsif.2014.0274 PMC4032540

[10] I. H. Chen , W. Yang , M. A. Meyers , Acta Biomater. 2015, 28, 2. 26391496 10.1016/j.actbio.2015.09.023

[11] a) A. R. Studart , Adv. Funct. Mater. 2013, 23, 4423;

[12] a) Z.‐L. Yu , N. Yang , L.‐C. Zhou , Z.‐Y. Ma , Y.‐B. Zhu , Y.‐Y. Lu , B. Qin , W.‐Y. Xing , T. Ma , S.‐C. Li , H.‐L. Gao , H.‐A. Wu , S.‐H. Yu , Sci. Adv. 2018, 4, eaat 7223; 10.1126/sciadv.aat7223 PMC6086613 30105307

[13] a) L. A. Berglund , I. Burgert , Adv. Mater. 2018, 30, e1704285; 29468736 10.1002/adma.201704285

[14] a) M. Frey , D. Widner , J. S. Segmehl , K. Casdorff , T. Keplinger , I. Burgert , ACS Appl. Mater. Interfaces 2018, 10, 5030; 29373784 10.1021/acsami.7b18646

[15] a) J. Song , C. Chen , C. Wang , Y. Kuang , Y. Li , F. Jiang , Y. Li , E. Hitz , Y. Zhang , B. Liu , A. Gong , H. Bian , J. Y. Zhu , J. Zhang , J. Li , L. Hu , ACS Appl. Mater. Interfaces 2017, 9, 23520; 28661650 10.1021/acsami.7b06529

[16] L. Burns , A. Mouritz , D. Pook , S. Feih , Compos. Struct. 2012, 94, 995.

[17] X. Wang , T. Keplinger , N. Gierlinger , I. Burgert , Ann. Bot. 2014, 114, 1627. 25180290 10.1093/aob/mcu180 PMC4649688

[18] R. Weinkamer , P. Fratzl , Mater. Sci. Eng., C 2011, 31, 1164.

[19] C. Mattheck , Interdiscip. Sci. Rev. 1994, 19, 298.

[20] U. Müller , W. Gindl‐Altmutter , J. Keckes , Trees 2018, 32, 1.

[21] K. Tu , L. Kong , X. Wang , J. Liu , Holzforschung 2016, 70, 1039.

