# Supplementary material for: Tunable Wood by Reversible Interlocking and Bioinspired Mechanical Gradients
Source: Adv Sci (Weinh). 2019 Mar 28;6(10):1802190. doi: 10.1002/advs.201802190 (PMC6524091; doi:10.1002/advs.201802190)
Supplement: Supplementary file 1 — Supplementary [file ADVS-6-1802190-s001.pdf]

Copyright WILEY-VCH Verlag GmbH & Co. KGaA, 69469 Weinheim, Germany, 2019.

ADVANCED SCIENCE
Open Access

Supporting Information

for *Adv. Sci.,* DOI: 10.1002/advs.201802190

Tunable Wood by Reversible Interlocking and Bioinspired Mechanical Gradients

Marion Frey, Giulia Biffi, Maria Adobes-Vidal, Meri Zirkelbach, Yaru Wang, Kunkun Tu, Ann M. Hirt, Kunal Masania, Ingo Burgert, and Tobias Keplinger**

Copyright WILEY-VCH Verlag GmbH & Co. KGaA, 69469 Weinheim, Germany, 2019.

Supporting Information

Tunable Wood by Reversible Interlocking and Bioinspired Mechanical Gradients

Marion Frey*, Giulia Biffi, Maria Adobes-Vidal, Meri Zirkelbach, Yaru Wang, Kunkun Tu, Ann M. Hirt, Kunal Masania, Ingo Burgert, Tobias Keplinger*

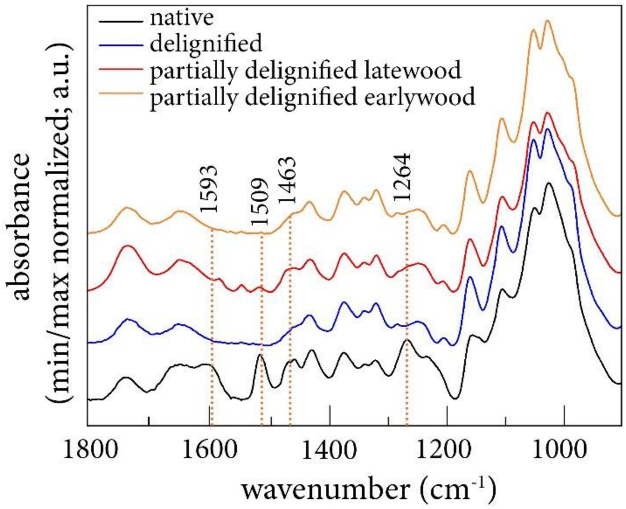

S1. FTIR spectra of native, delignified, partially delignified latewood and partially delignified earlywood in the spectral range from 1800 to 900 cm^{-1}. The characteristic lignin peaks at 1593, 1463, 1509 cm^{-1} (aromatic skeletal vibration) and 1264 cm^{-1} (guaiacyl ring breathing with CO stretching) (Schwanninger, 2011[1]) are present in native wood and disappear upon delignification. Partially delignified wood shows lignin signals mainly in the latewood region, whereas the earlywood is completely delignified.

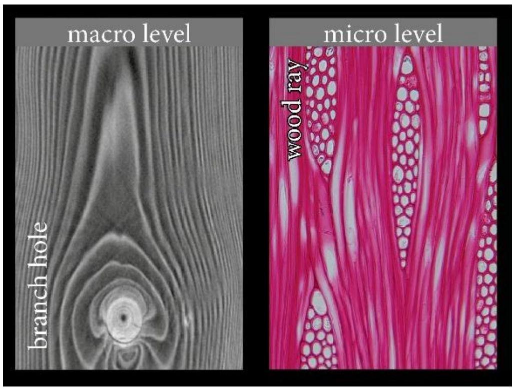

S2. Bioinspiration from trees on macro level (branch hole) and on micro level (wood ray)

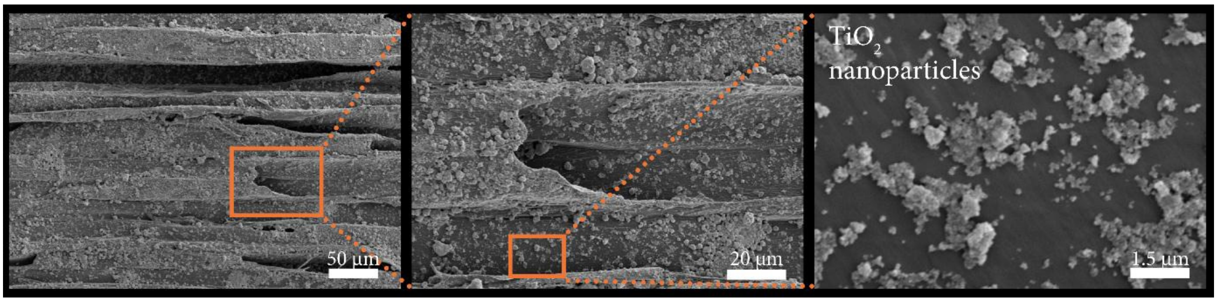

S3. SEM images of the superhydrophobic TiO_2/PDMS coating on a delignified veneer surface (left) and zoom into regions of interest (middle, right).

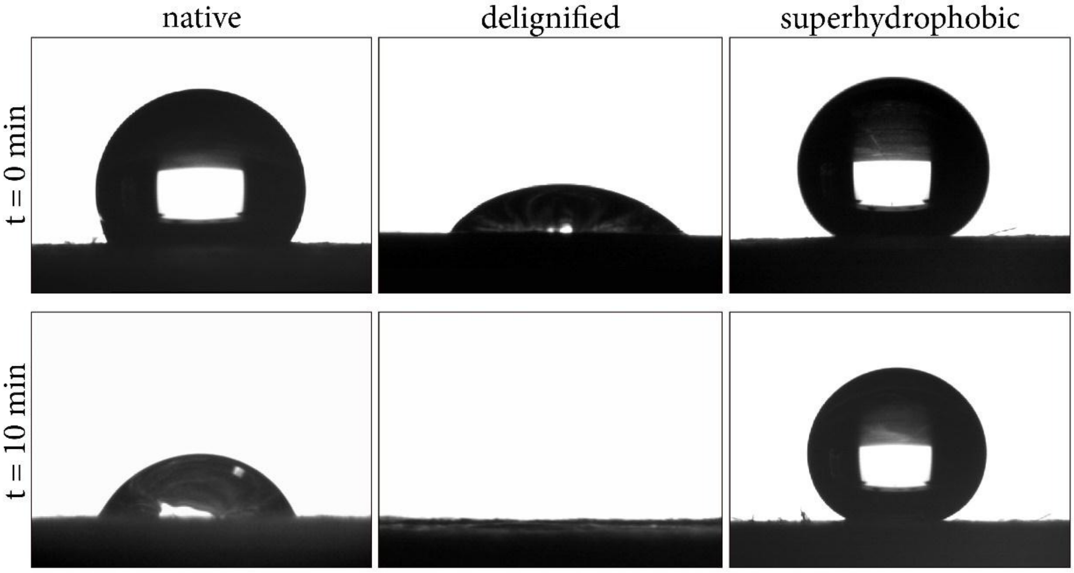

S4. Contact angle measurements of native, delignified and delignified/superhydrophobic wood measured after t = 0 min and t = 10 min. The contact angle on native and delignified wood changes with time, whereas the droplet on the superhydrophobic surface keeps its shape.

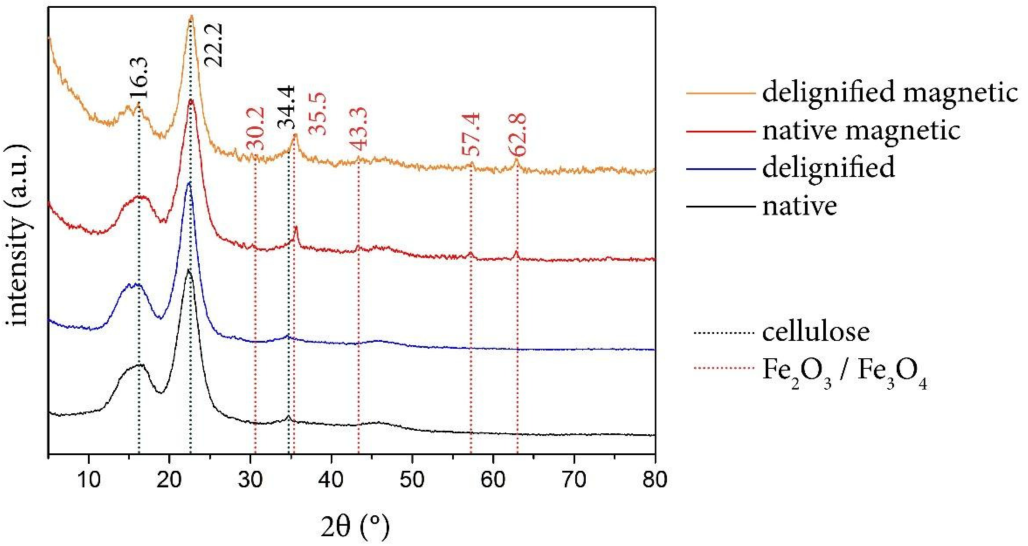

S5. XRD pattern of native, delignified and native- and delignified magnetic wood. The characteristic crystalline cellulose peaks appear at 16.3, 22.2 and 34.4° (Park, 2010[2]). Maghemite (Fe_2O_3) or magnetite (Fe_3O_4) show characteristic peaks at 30.2, 35.5, 43.3, 57.4 and 62.8° (Schimanke et al., 2000[3]).

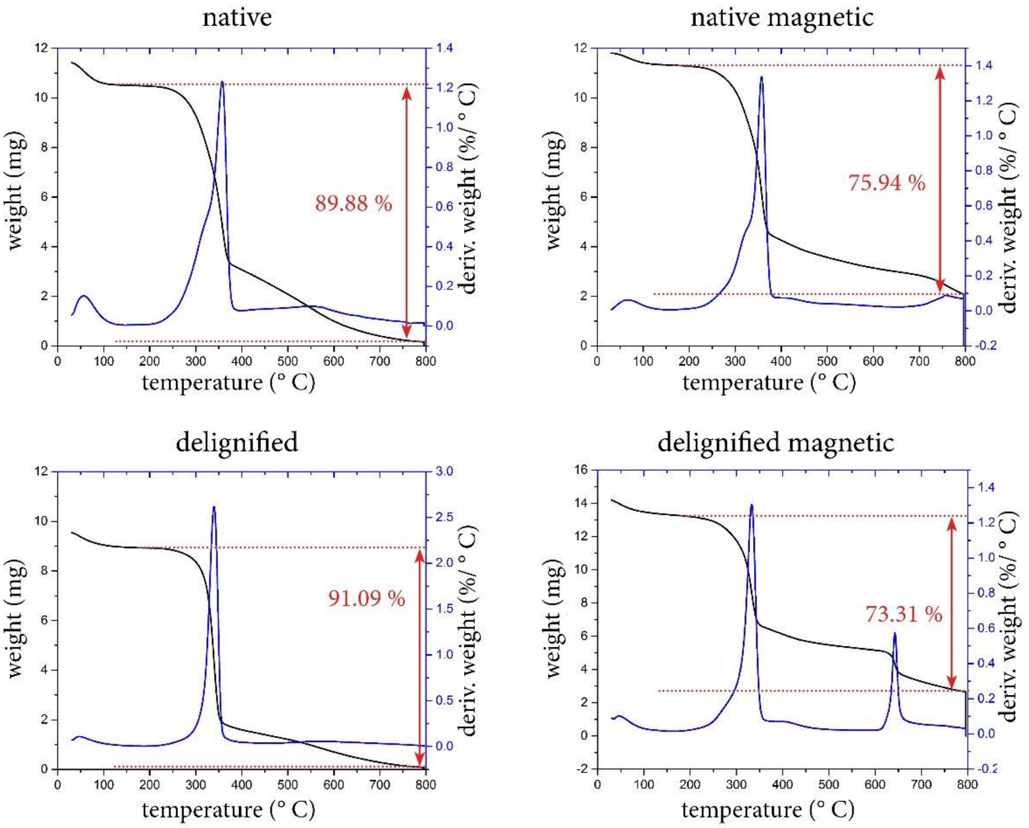

S6. Representative TGA curves for native, native magnetic, delignified and delignified magnetic wood. A weight gain of approximately 15% was obtained upon magnetization.

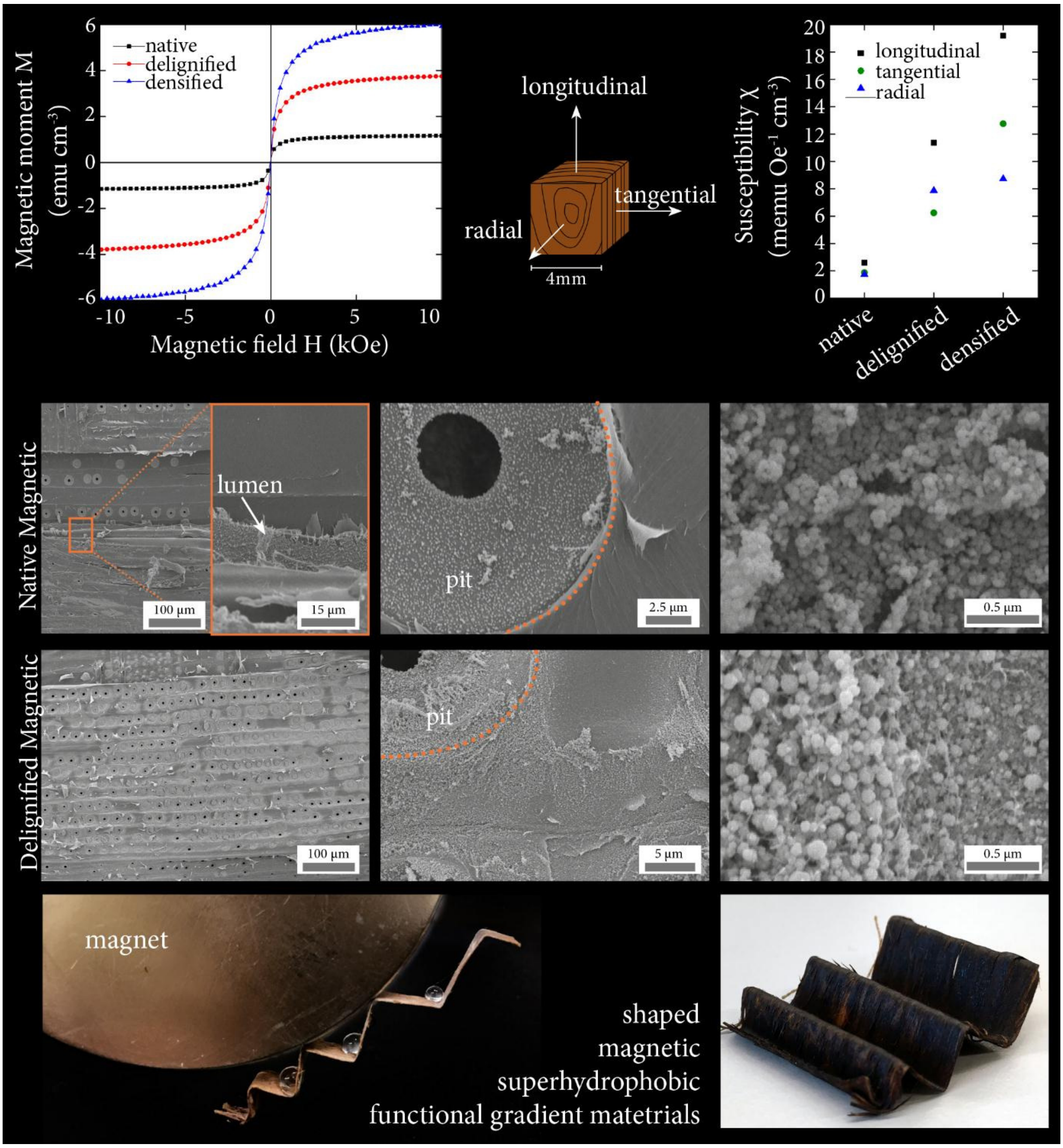

S7. (a) Magnetization behavior of native, delignified and delignified/densified magnetic samples and (b) susceptibility in longitudinal, tangential and radial direction. (c) SEM images of longitudinal cuts of native magnetic wood. (d) SEM images of delignified magnetic wood. (e) Shaped, magnetic, superhydrophobic wood, which is attached to a magnet. (f) Functional gradient material showing a higher magnetic moment and higher density on the right side (3 layers) than on the left side (1 layer).

Supplementary methods

	dimensions r x t x l [mm^3]	1st delignification time at 80 °C [hours]	2nd delignification time at 80 °C [hours]
bulk	100 x 10 x 20	6	6
bulk (thin)	100 x 5 x 20	6	4
veneer delignified	150 x 90 x 0.9<br>150 x 90 x 1.5	6	-
veneer partially delignified	150 x 90 x 0.9	1	-
cubes	5 x 5 x 5	6	-

Table S1: Delignification time according to the sample dimensions. Bulk samples were delignified a second time after exchanging the reaction solution.

Preparation of specimens with varying fiber volume content. Thin bulk samples (conditioned at 95% RH, 20 °C) were unidirectionally stacked and densified to a thickness of 5 mm using a closed mold following our previously established protocol.[4] Adhesive (Loctite Purbond HBS309) was applied in between the individual layers on the very top and bottom part of the samples, in order to avoid shear movements between layers in the clamped regions during mechanical testing. The test region was free of adhesive to ensure a matrix/adhesive independent characterization of the mechanical properties. Beech tags (2 mm thick) with an angle of 45 ° were glued to the grip section to avoid damage upon clamping in the universal testing machine.

Superhydrophobization. TiO_2 (Titanium (IV) oxide, anatase, <25 nm in size), Trichloro (1H, 1H, 2H, 2H-perfluorooctyl)silane ($CF_3(CF_2)_5CH_2CH_2SiCl_3$, PFOTS) were purchased from Sigma-Aldrich (Buchs SG, Switzerland). Polydimethylsiloxane (PDMS, Sylgard 184) and the corresponding curing agent were obtained from Dow Corning (Auburn, MI, USA). Tetrahydrofuran (THF, ≥99.0%) was supplied by VWR International (Zurich, Switzerland). All chemicals were used as received. The reaction protocol was adapted from Tu et al., 2016.[5]

In short, TiO_2 (1 g), THF, (60 ml) and PFOTS (0.5 ml) were mixed in a 150 ml beaker. The mixture was stirred at 65°C for 4 h under constant stirring and then dispersed with an ultrasonicator for 10 min. The PFOTS-modified TiO_2 particles were then collected by centrifugation and finally re-dispersed in 60 ml THF for further use. PDMS precursor (1 g) was dissolved in THF (30 ml) under magnetic stirring. PFOTS-modified TiO_2 nanoparticles were then dispersed in the PDMS solution under ultrasonication for 20 min to obtain solution A. PDMS curing agent (0.1 g) dissolved in THF (15 ml) results in solution B. Just prior to the coating treatment, solution A and B were mixed under ultrasonic dispersion for 20 min to form the PDMS/TiO_2 coating solution. The delignified wood samples were dipped into the coating solution for 10 seconds and dried at 65°C for 30 min. This procedure was repeated 3 times and the coated samples were then cured at 65°C for 5 h.

Magnetization. In situ magnetization of native and delignified wood cubes (5 x 5 x 5 mm^3) was conducted via coprecipitation of ferric and ferrous ions to obtain iron oxide particles as described in detail by Merk et al., 2014.[6] Prior to modification, the samples were dried at 65 °C and a Teflon tape was tied around the wood samples to keep their structural integrity during modification. The following two solutions were prepared for the modification: Solution A: Ferric and ferrous chloride suspension with a fixed molar ratio of 2:1 with the

concentrations 1.32 mol l^{-1} ferric chloride ($FeCl_3$) and 0.66 mol l^{-1} ferrous chloride ($FeCl_2$). Solution B: 25 ml of 25% aqueous ammonia was added to 400 ml deionized water.
The samples were vacuum infiltrated with solution A 3 times for 6 h. The samples were afterwards transferred to solution B for having a rapid addition of excess ammonia and remained in the solution for 24 h. Magnetic samples were washed with deionized water after the treatment until a neutral pH value of the washing solution was reached.

Microtome and Ultramicrotome Cutting. Sample cross-sections analyzed by light-microscopy (Olympus BX51) and AFM (JPK Instruments AG) were prepared using a rotary microtome (Leica Ultracut, Germany). Additionally, samples used for AFM measurements and shaped samples embedded in epoxy (Epofix) were cut using an ultramicrotome (Reichert-Jung Ultracut, Germany) with a diamond knife (Diatome).

Atomic Force Microscopy (AFM). AFM imaging was performed on a Nano Wizard 4 (JPK Instruments AG, Germany) at 20 °C and 65% relative humidity.
In situ measurements of the cell corner of delignified spruce (Figure 2) were conducted in a petri dish filled with 10 mL of unstirred deionized water in contact mode. Cantilevers with a silicon probe and a nominal spring constant of 0.3 N m^{-1} (CONTR, Nano world, resonance frequency 15 kHz) were used. The 10 x 10 μm^2 images were obtained at a line scan rate of 0.7 Hz and set points between 1 and 6 nN with a resolution of 512 x 512 pixels. AFM images of the cell corner before and after the submersion in water were acquired using the same imaging parameters.
The measurements of densified cell walls (Figure 3) were conducted in the quantitative imaging mode with a resolution of 256 x 256 pixels and a scan size of 20 x 20 μm^2. A noncontact cantilever (NCHR, Nano World, resonance frequency 320 kHz) with a silicon probe was used with a set point of 60 nN, z-length of 100 nm and a pixel time of 12 ms. The thermal noise method was used for calibration of the cantilever. Images were processed with the JPK image processing software (JPK Instruments AG).

Water content. The gravimetric water content of the locked, intermediate and morphing state was calculated by dividing the mass of water in the conditioned sample by the mass of the oven-dry sample. The mass was measured after conditioning at 20 ˚C/65% RH (locked state), 20 ˚C/95% RH (intermediate state) and after delignification and washing for obtaining the water content in wet (morphing) state. Samples were oven dried at 103 ˚C for obtaining the dry mass. 3 samples per condition were tested.

Tensile testing. Tensile tests were performed on a universal testing device (Zwick Roell, Germany) equipped with a 100 kN load cell. Conditioned specimens (25°C 65% RH) with the dimensions of 100 x 5 x 10 mm^3 were tested with a span length of 36 mm, and the displacement was measured with a travel sensor with an initial length of 20 mm. The tests were conducted at 20 °C and 65 % relative humidity and a speed of 5 mm min^{-1} and 6 samples per condition were tested.

Scanning Electron Microscopy (SEM). Samples were coated with a sputter coater (CCU-010, Safematic). A Pt-Pd (80/20) coating of ~6 nm thickness was applied and the samples were analyzed with a Hitachi SU 5000 equipped with a SE detector.

Contact angle measurements. Static contact angles (CAs) were measured with the sessile drop method on tangentially cut wood veneers (native, delignified, modified). A Drop Shape Analyzer-DSA100 (Krüss GmbH) was used at ambient condition with a water droplet volume of 8 µl. The contact angle was determined immediately after contact and in constant time

intervals up to 10 minutes. Time intervals of 80 s were used for the native and the superhydrophobically treated delignified wood. For delignified wood, the interval was set to 12 s in the beginning.

Vibrating sample magnetometry. The magnetic hysteresis was measured with a MicroMag 3900 Vibrating Sample Magnetometer (Princeton Measurements Corporation). The applied magnetic field was ranging from -10 kOe to 10kOe in 100 Oe steps and an averaging time of 100 ms.

Kappabridge. A KLY-2 inductive susceptibility bridge (AGICO) was used to measure the susceptibility and the anisotropy of low-field magnetic susceptibility at a magnetic field of 200 A m^{-1}.

Fourier Transform Infrared Spectroscopy (FTIR). Wood veneer surfaces were analyzed with an ATR-FTIR spectrometer (Bruker Tensor 27). Baseline correction (concave rubber band) and normalization (min/max) of the obtained spectra was conducted in the software OPUS.

X-ray Diffraction (XRD). XRD diffraction patterns were recorded for native and delignified spruce and for the magnetically modified native and delignified wood cubes. The surface of a longitudinal cut was analyzed using a PANalytical Empyrean diffractometer (Almelo, Netherlands) in the Bragg-Brentano mode with a Cu Kα radiation. A 2θ range of 5 - 80° was measured. For magnetic samples, a nickel filter was used to reduce the fluorescence. The diffraction curves were normalized and smoothened in OriginPro 2016.

Thermogravimetric analysis (TGA). Wood cubes were cut into small pieces with a razor blade and were analyzed with a TGA under nitrogen atmosphere. A TGA Q50 (TA Instruments) with a heating rate of 10 C min^{-1} (30-800 °C) was used for the measurement.

References

[1] M. Schwanninger, J. C. Rodrigues, K. Fackler, J. Near Infrared Spectrosc. **2011**, 19, 287.

[2] S. Park, J. O. Baker, M. E. Himmel, P. A. Parilla, D. K. Johnson, Biotechnol. Biofuels **2010**, 3, 10.

[3] G. Schimanke, M. Martin, Solid State Ionics **2000**, 136, 1235.

[4] M. Frey, D. Widner, J. S. Segmehl, K. Casdorff, T. Keplinger, I. Burgert, ACS Appl. Mater. Interfaces **2018**, 10, 5030-5037.

[5] K. Tu, L. Kong, X. Wang, J. Liu, Holzforschung **2016**, 70, 1039.

[6] V. Merk, M. Chanana, N. Gierlinger, A. M. Hirt, I. Burgert, ACS Appl. Mater. Interfaces **2014**, 6, 9760.
